# Internal Communication and Dissemination Strategies During Magnet Implementation in German Hospitals: A Qualitative Study

**DOI:** 10.1155/jonm/3517120

**Published:** 2026-01-04

**Authors:** Joan Kleine, Carolin Gurisch, Julia Köppen, Reinhard Busse, Lisa Smeds Alenius

**Affiliations:** ^1^ Department of Healthcare Management, Technische Universität Berlin, Berlin, Germany, tu-berlin.de; ^2^ Department of Health Sciences, BQS Institute for Quality and Patient Safety GmbH, Hamburg, Germany; ^3^ School of Public Health, Bielefeld University, Bielefeld, Germany, uni-bielefeld.de; ^4^ Department of Learning, Informatics, Management and Ethics, Karolinska Institutet, Stockholm, Sweden, ki.se

**Keywords:** dissemination, hospital, implementation, internal communication, Magnet, Magnet4Europe, nursing, organizational change

## Abstract

**Aim:**

To explore internal strategies for communicating and disseminating the implementation of Magnet principles in German hospitals from the perspective of implementation leaders.

**Design:**

A qualitative study.

**Methods:**

Between January 2023 and June 2024, 15 semistructured interviews were conducted with 16 participants from six German hospitals participating in the Magnet4Europe project. Data were analyzed using qualitative content analysis, following the COREQ guidelines.

**Results:**

Four major themes emerged: (i) communicating through multiple channels, (ii) choosing effective wording, (iii) leveraging enabling factors for dissemination, and (iv) navigating dissemination challenges. The combination of visual, written, and verbal communication channels, as well as consistent and tailored language, was seen as essential for communicating the implementation of Magnet. Leadership support, contextual adaptation of terminology, and linking Magnet principles to practical applications were perceived as facilitating acceptance. Challenges included difficulties in reaching all target audiences and overcoming staff resistance.

**Conclusion:**

Communication was perceived as playing a vital role in supporting the implementation and dissemination of Magnet and in fostering efforts to embed Magnet principles within the organizational culture.

**Practical Implications:**

Effective dissemination of Magnet requires a multifaceted communication approach that combines visual, verbal, and interpersonal strategies. Leadership support, tailored language, staff engagement, and continuous communication were identified as key elements for embedding Magnet principles into daily hospital practice and fostering long‐term cultural change.

## 1. Introduction

The working conditions in nursing across European hospitals are characterized by a high workload and shortage of skilled personnel. This leads to high burnout rates, job dissatisfaction, and nurses leaving the profession [1–3]. Optimizing the work environment is crucial to promote staff well‐being, improve patient care, and make the nursing profession more attractive [3–5].

Effective communication within hospitals plays a pivotal role in addressing these challenges. High‐quality communication is essential for aligning organizational goals with staff needs, reducing uncertainty, and improving job satisfaction during periods of change [6, 7]. In the context of systemic improvements, communication strategies ensure that all levels of staff are informed, engaged, and empowered to contribute to organizational transformations [7].

## 2. Background

The Magnet model offers a strategic framework for improving hospital work environments. Originating in the 1980s, it was developed based on the characteristics of hospitals from the United States that successfully recruited and retained nurses during a national nursing shortage [8]. The Magnet model consists of five key principles: (i) transformational leadership, (ii) structural empowerment, (iii) exemplary professional practice, (iv) new knowledge, innovations, and improvements, and (v) empirical outcomes [9, 10]. To achieve Magnet designation, hospitals must fulfill all five Magnet key principles, which entail a significant organizational transformation.

A systematic review has examined the impact of Magnet in Anglo‐American settings, finding increased patient satisfaction, improved clinical outcomes, lower rates of job‐related burnout, and greater success in recruiting and retaining healthcare professionals compared to non‐Magnet hospitals [10].

Despite recurring and consistently positive results in U.S. hospitals, as of May 2024, approximately 98% of Magnet‐designated hospitals remain in the United States, with only a limited number established internationally [11].

From January 2020 to June 2024, the EU‐funded project Magnet4Europe (M4E) took place and aimed at exploring the implementation of the principles of Magnet in over 60 European hospitals from Belgium, England, Germany, Ireland, Norway, and Sweden [12]. The overarching goal of M4E was to improve clinicians’ mental health and well‐being through changes in workplace culture in European hospitals [12]. The intervention was supported by one‐to‐one twinning between European hospitals and U.S. Magnet‐designated hospitals, international learning collaborations, national hospital networks, feedback reports based on the online surveys of nursing and medical staff, and receiving the Magnet Application Manual [12–14].

In Germany, a total of 20 hospitals participated in M4E, with 18 remaining actively involved until its conclusion [15]. However, to date no hospital in Germany has been Magnet designated, and there is currently no evidence of a fully realized implementation of the model in the German healthcare system [16]. Participating M4E hospitals were autonomous in how they chose to implement the Magnet principles set out in the Magnet Application Manual [13] and what components were deemed relevant for their particular organization. Pursuing or achieving Magnet designation was not part of the study nor a prerequisite for participation. In the following, the term “Magnet” refers to the principles and their application in the M4E project. In the German hospital context, traditional hierarchical structures, pronounced profession‐specific boundaries, and limited experience with participatory leadership approaches such as shared governance may pose challenges to the adoption of Magnet [17, 18].

Svensson et al. [19] examined the early phase of implementing Magnet in eleven European acute care hospitals participating in M4E. The results showed that during this phase, the implementation leaders primarily focused on creating a supportive space for discussion and co‐design with nursing staff to foster their acceptance and engagement. Key to this process was understanding and interpreting Magnet to adapt it to the local context of each hospital. The implementation leaders had to translate the principles in a way that made them relevant to the specific circumstances and challenges of their institutions [19]. These findings provide a valuable basis for exploring approaches and factors influencing the communication and dissemination of Magnet in German hospitals.

Implementing Magnet requires a cultural shift toward patient‐centeredness and employee orientation, ultimately aiming to sustainably improve the clinical work environment [9]. In some of the participating German hospitals, this shift included measures such as introducing shared governance structures and strengthening the role of nursing leadership.

Implementation processes in healthcare are increasingly understood as complex, nonlinear, and context‐dependent. The i‐PARIHS framework conceptualizes successful implementation as the dynamic interaction of evidence, facilitation, and context, with a strong emphasis on relational processes such as communication, negotiation, and adaptation [20]. This perspective reinforces the understanding that communication is not merely a technical function but a core mechanism in enabling change. In line with implementation science literature, communication supports sense‐making, participation, and cultural adaptation [6, 21, 22], making it a key component of implementation efforts aimed at transforming organizational culture.

New work area structures introduced during hospital restructuring can lead to negative expectations and concerns among employees, particularly related to insufficient staffing and potential disruptions to workflows, as shown in a study from Sydney, Australia [23]. Concurrently, the study also reported positive expectations regarding patients’ benefits and staff adaptability [23].

Ellis et al. [6] further examined the role of communication and team culture in supporting staff readiness for change in a hospital undergoing major transformation in Sydney, Australia. Their cross‐sectional study among clinical and nonclinical staff found that feeling informed mediated the relationship between team culture and change readiness, which in turn was negatively associated with burnout. Communication was thus not only instrumental but also a mechanism to foster change readiness and resilience in complex health settings [6, 22].

### 2.1. The Study

This study aims to investigate German hospitals’ strategies to communicate and disseminate the implementation of Magnet principles, focusing on the perspective of those leading the implementation process. The objectives are to explore the channels and language used to inform staff about Magnet and to understand enablers and barriers within the communication strategies used for the internal dissemination of complex change initiatives such as Magnet implementation.

## 3. Methods

### 3.1. Study Design

This study followed a qualitative approach and was conducted as part of the M4E project. The data collection was part of the process evaluation in Germany, examining organizational processes, contextual factors, and barriers and facilitators in implementing Magnet in participating hospitals [12, 19]. Semistructured expert interviews were conducted, following the consolidated criteria for reporting qualitative research (COREQ) [24] (Table A1).

### 3.2. Study Setting and Recruitment

Six German hospitals were included in this study. First, purposive sampling was used to select two M4E hospitals with the highest and lowest burnout scores [19], as measured in the baseline staff survey using the Burnout Assessment Tool [25]. This aimed to capture potential differences in motivation and ambition among the hospitals. Second, four additional M4E hospitals were chosen through convenience sampling. This ensured the inclusion of case hospitals with diverse organizational characteristics, such as university hospitals, specialist clinics, and maximum‐care providers, offering additional perspectives on the implementation processes in Germany.

Purposeful sampling identified two to three key individuals per hospital holding leadership roles in implementing Magnet through the M4E project. These individuals were typically involved in project coordination or sponsorship, which included nurse managers, nursing scientists, Chief Nursing Officers (CNO), physicians, and quality managers. Their perspectives provided in‐depth insight into strategic considerations and communication practices during the change process. Participants were contacted by a member of the German M4E research team, provided with detailed written information, and invited to discuss the implementation process.

### 3.3. Data Collection

A total of 15 semistructured interviews were conducted with 16 individuals between January 2023 and June 2024, either online or in person. The interviews took place approximately 2.5–3.5 years after the start of the M4E intervention in the hospitals. In one hospital, two individuals were interviewed together due to time constraints. Interviews lasted between 37 and 82 min. After obtaining written informed consent, the interviews were audio‐recorded. Sample size was determined using the concept of Information Power [26], which suggests that smaller sample sizes are sufficient when the collected information is highly specific.

The semistructured interview guide used in this study consisted of thematic sections covering personal experiences with the Magnet model, the implementation process of the underlying principles, and perceived facilitators and barriers. Specific questions addressed leadership roles, team involvement, and the adaptation of Magnet to the local hospital context. Probing questions were included to deepen insights and clarify responses, ensuring a comprehensive understanding of the implementation dynamics, e.g., for understanding the communication and dissemination of Magnet.

### 3.4. Data Analysis

The recorded interviews were transcribed verbatim, and all potentially identifiable information was removed and replaced with pseudonyms. Coding was conducted using the software Atlas.ti, based on Mayring’s qualitative content analysis and a deductive‐inductive approach to identify relevant interview material for answering the research question as openly as possible [27].

The analysis of the transcripts followed an iterative and collaborative process to ensure precise and valid coding of the data in three main steps. In the first step, one of the authors and a second coder familiarized themselves independently with one‐third of the interview material (five transcripts), focusing on relevant text segments regarding predefined codes of (i) strategies of communicating the implementation of Magnet and (ii) factors influencing its dissemination. In the second step, this material was then further analyzed in‐depth inductively. The resulting insights were refined through discussion and used to develop a category system. In the third step, the entire interview material was divided between the two team members and coded according to the developed category system. Throughout this process, interim results were regularly compared and discussed to identify similarities and discrepancies and to further refine the coding. The coding tree is provided in Table A2.

### 3.5. Ethical Considerations

The study was approved by the Ethics Committee of Charité–Universitätsmedizin Berlin (No. EA1/243/20). Interviewees were informed about the study’s objectives and background, the voluntary nature of participation, and data protection both prior to and on the day of the interview. All interviewees provided written consent. Consent could be withdrawn at any time without consequences until the data were transcribed and anonymized, but no one chose to do so. The results and quotes were anonymized to protect the identity of the interviewees. Additionally, no specific details about the hospitals where the interviewees worked were included, ensuring the anonymity of both the participants and their respective hospitals.

### 3.6. Rigor and Reflexivity

Several measures were applied to ensure rigor and reflexivity. This involved researcher triangulation whereby multiple researchers were involved in the data collection, analysis, and interpretation of the study. Quality was further maintained by engaging two researchers to reduce bias during coding and interpretation. Regular discussions about coding were held with two more researchers to ensure diverse perspectives, enhancing the reliability and validity of findings [28]. Researchers also documented their assumptions and reflections throughout the study to acknowledge potential influences on the research process. The study adhered to the COREQ guidelines, promoting transparency in data collection and analysis [24]. The dependability and transferability of this study are supported by a thorough description of the methodology as well as the presentation of interview quotes and their associated codes.

## 4. Results

### 4.1. Sample Description

The size of the hospitals ranged from approximately 150 to 1300 beds. Of the 16 interviewees, ten were female and six were male. A total of eight interviewees served as lead or co‐managers or coordinators of the project. Four interviewees were CNO and acted as project sponsors, e.g., representing members of an M4E steering group and/or the hospital executive board. Another four interviewees were identified as members of the hospitals’ M4E project teams (see Table 1).

**Table 1:** Interviewee characteristics.

Hospital (H)	H1	H2	H3	H4	H5	H6	Total
Number of participants	3	3	2	2	3	3	16
Female	2	1	2	1	3	1	10
Role in the M4E implementation process:							
Project manager or project coordinator	1	2	2	1	1	1	8
Project sponsor, e.g., member of a M4E steering group and/or hospital executive board	/	1	/	1	1	1	4
Member of hospital’s M4E project team	2	/	/	/	1	1	4

Abbreviations: H, hospital; M4E, Magnet4Europe.

### 4.2. Main Themes and Subthemes

All 15 transcripts were included in the analysis, regardless of whether direct quotes are shown in the following. To address the study’s aim of exploring strategies to communicate and disseminate the Magnet implementation in German hospitals, the analysis of the interviews identified four main themes and eleven subthemes: (i) communicating through multiple channels, (ii) choosing effective wording, (iii) leveraging enabling factors for dissemination, and (iv) navigating dissemination challenges of Magnet. Table 2 provides an overview of the main themes and subthemes.

**Table 2:** Overview of main themes and subthemes.

Main themes	Subthemes
Communicating through multiple channels	Visual and written media
	Meetings, team training, and events
	Use of ambassadors

Choosing effective wording	Use of the term “Magnet”
	Tailoring of terminology

Leveraging enabling factors for dissemination	Leadership support
	Staff involvement
	Continuity of communication
	Establishing practical relevance

Navigating dissemination challenges	Difficulties in reaching the target audience
	Staff resistance

### 4.3. Communicating Through Multiple Channels

The interviewees described using multiple communication channels to convey Magnet across the hospitals. Visual and written tools were used to enhance awareness and provide detailed information, while verbal communication and personal engagement, including ambassador teams, were highlighted as crucial for fostering deeper understanding and involvement with Magnet.

#### 4.3.1. Visual and Written Media

In all hospitals, interviewees reported the use of various visual and written media to inform about Magnet. This included print media such as posters, banners, and flyers, as well as digital media like intranet, newsletters, and social media. A combination of these communication channels was used to achieve the widest possible reach, as expressed by one interviewee:

*“The reach is always a big issue. You try to reach as many people as possible through different ways, be it newsletters on the intranet, social media on the hospital’s website […], through One Minute Wonder [poster], […], where the topic is brought up repeatedly.” (ID10)*

According to several interviewees, posters and banners were specifically used to increase the visibility of Magnet. These visual materials were distributed across the wards to draw staff attention. Banners complemented the posters and were also placed in various locations.

The usage of a specific format of posters, known as ‘One Minute Wonder,’ was described by interviewees from two case hospitals. These posters contained short and concise information on the M4E project, the Magnet model itself, and/or various other related topics, designed to be easily understood and quickly absorbed. These posters were placed in areas where staff would pause, such as hallways, outside examination rooms, break rooms, near blood gas analyzers, or on the inside of restroom doors. The posters were created by the staff themselves.

Interviewees noted that the hospitals’ intranet served as a central information platform for Magnet. In one hospital, there was a dedicated intranet page specifically for Magnet, where staff could find detailed information about the implementation of the project and opportunities for involvement.

#### 4.3.2. Meetings, Team Training, and Events

The interviewees described how visual and written media were complemented by verbal and personal communication, which played a central role in the dissemination of Magnet in all hospitals. Interviewees mentioned various occasions where this was used, such as interdisciplinary events, the integration of Magnet into team and staff meetings, and the organization of special events.

Interdisciplinary leadership training was mentioned as an important component of verbal communication. Both future senior doctors and nurses preparing for leadership positions participated in these trainings. The implementation of Magnet was firmly integrated into these training courses and presented as a standard part of leadership responsibilities.

*“We have leadership training sessions […]. And there’s a large interdisciplinary project where we bring top leaders from all professions, future senior doctors, and nurses preparing for a director of nursing position. […] In this leadership training, the presentation of the Magnet project and how it connects to leadership roles is already standard.” (ID4)*

The integration of Magnet into regular team meetings was identified by several interviewees as another key communication strategy. It was reported that Magnet was discussed at leadership retreats and regular meetings with all ward managers. The minutes from these meetings were distributed to all wards to ensure broad information dissemination.

*“We discussed it [Magnet] in the leadership retreat for two days. It’s part of every team meeting with all ward managers, […] monthly. These minutes are sent to the wards, and it’s included there.” (ID13)*

In addition to utilizing existing structures for communication about Magnet, some interviewees mentioned specific actions or events designed to make the implementation project of Magnet more visible and promote staff interest and involvement. One example of an event was the organization of celebrations on appropriate days, such as International Nurses Day. These celebrations not only served to recognize and appreciate nursing staff but also to promote Magnet. These actions included information booths where staff were informed about Magnet directly and initiatives such as distributing small gifts to staff on the wards. Such actions helped draw staff’s attention to Magnet and spark interest.

*“So, we participate in many events where we always have a Magnet booth to provide information. We were at Patient Safety Day, and for the first time this year, we celebrated and organized something on Nurses Day. And we keep sprinkling in Magnet information here and there, which definitely piques interest, with people asking; what is this.” (ID7)*

#### 4.3.3. Use of Ambassadors

The use of ambassador teams as multipliers was highlighted as an important element of verbal communication across all case hospitals. Although interviewees in different case hospitals described slightly different approaches to the selection and use of ambassadors, these teams consisted of staff from various professions who acted as advocates for Magnet and shared information within their respective teams and departments. One interviewee mentioned that establishing ambassador teams was considered a milestone for better integrating staff nurses into Magnet implementation.

*“We set up the multiplier group. I think that was a very important milestone in better integrating nursing practice.” (ID5)*

In one hospital, the ambassador concept was still in the stage of planning. The hope was that the ambassadors would actively promote Magnet and explain the significance of the implementation to staff in a more accessible way.

*“That’s why we want to recruit these multipliers, hoping they will become positive advocates for the Magnet project and explain to the staff, in even better words, what it really means, because, in the end, it’s not rocket science what we’re doing.” (ID14)*

It was also reported that members of the project group themselves acted as ambassadors and played a central role in disseminating Magnet. Through their positions and involvement, they shared information within their respective teams and acted as intermediaries between project coordination and staff. This was described as an especially effective method for achieving broad acceptance and participation.

*“Through the project group, which included people from various positions, we had a kind of multipliers, with information being passed on to their teams and colleagues. That worked very well […].” (ID10)*

### 4.4. Choosing Effective Wording

According to the interviewees, effective communication required careful consideration of language. This included both the deliberate use of the term “Magnet” in internal communication as well as the adaptation of terminology to the German context to make the underlying principles of the Magnet model more accessible to staff.

#### 4.4.1. Use of the Term “Magnet”

According to all interviewees, the term “Magnet” was deliberately used in internal communication. Rather than merely being a label, Magnet was associated with a structured framework and a clear set of requirements. Using this term helped to position Magnet‐related initiatives as part of a strategic and well‐defined approach to improving the hospital environment. The interviewees described that the term provided a recognizable reference point, making it easier to communicate overarching goals and align various projects under a common vision.

*“The great thing about Magnet is that […] it provides a framework and a roof, along with a set of requirements, which allows you to get things through that you otherwise wouldn’t. And for that, it’s essential to use the word Magnet.” (ID4)*

It was also reported that the term “Magnet hospital” was regularly used to emphasize the involvement in the M4E project. Over time, it became ingrained in the staff’s vocabulary, reinforcing the association with positive organizational changes and increasing recognition and acceptance within the hospital environment.

*“Yes, we use the term Magnet hospital and that’s something that, as I mentioned earlier, has already become ingrained as a term.” (ID5)*

One interviewee initially expressed concerns that the term “Magnet” might be oversimplified. However, it was later observed that over time it became a synonym for positive changes and resource provision. The interviewees noted that staff associated it over time with beneficial projects and improvements, which had increased acceptance and support for the program.

*“At the beginning, I thought […] you reduce something like that to a single term. But that term has become a synonym. And that promotes […] projects and resources, and the provision of funds, when people associate it with something positive, and I think that’s already happening here.” (ID5)*

Other interviewees reported that the term “Magnet” was not always received positively. Some staff understood it as an external certification process and associated it with additional bureaucratic effort without clear benefits for them. However, the communication strategy was maintained to keep activities clearly aligned under the “Magnet project” and emphasize its strategic importance, which, according to some interviewees, contributed to a coherent and focused implementation.

*“We noticed that it’s not always perceived positively everywhere, and we’ve now focused in the communication more on saying, this is the Magnet project.” (ID13)*

#### 4.4.2. Tailoring of Terminology

Although the term “Magnet” was predominantly used for communication, some terms and explanations from the Magnet model were difficult to understand or uncommon in the German language. Particularly, older staff members showed resistance to anglicisms. Therefore, one interviewee suggested that the focus should not be on using the exact terms of the Magnet model, but rather on the goals of the model, such as improving the work environment and patient care.

*“What became clear to us very quickly is that we can’t work with these extreme terms like structural empowerment, transformational leadership, outcome, and professional practice. […] People can’t work with those terms, but we quickly realized that Magnet is primarily about bringing patient care back into focus.” (ID3)*

In one of the hospitals, terminology was initially adapted to make the Magnet model more understandable for staff. This involved finding appropriate German equivalents for the English terms and tailoring the content to the target audience. As understanding of the model grew among staff, the English terms were gradually introduced.

*“We’re trying to adapt the terms, and everything that is meant by them, to us. The model remains intact. […] The terms themselves are very cumbersome. And we need to […] find a language that is understandable for the staff. ‘Structural empowerment’ is not tangible for most. We’ve gone […] through a multi-step process and initially worked with questions to explain what is meant.” (ID13)*

### 4.5. Leveraging Enabling Factors for Dissemination

The interviewees reported that the dissemination of Magnet required a multifaceted approach, combining leadership support, practical relevance, active staff involvement, and continuous communication. These factors were seen as critical for ensuring the acceptance of Magnet and the implementation within the hospitals.

#### 4.5.1. Leadership Support

Interviewees described how the commitment and support of management and other leaders were crucial for the dissemination and acceptance of Magnet. Interviewees reported how staff were more likely to feel confident in raising new issues and pursuing changes when they were assured of their leaders’ support and commitment.

*“Because only when I know that they [the leaders] stand behind me and support me do I, as an employee, feel confident enough to address such issues and push for change, especially with projects I am not familiar with.” (ID3)*

Leaders who actively advocate for Magnet and motivate their teams were seen as beneficial for the project’s implementation, spread, and acceptance. One person shared a positive example of a leader who took time for personal conversations and celebrated work‐related milestones with colleagues.

*“[The leader] has all the conversations with staff, right there on the ward. […] She praises and celebrates a lot with the colleagues. She embodies much of what Magnet is about. […] and it works.” (ID4)*

Interviewees described how influential leaders could serve as role models and foster the project’s acceptance. As an example, one interviewee describes how a senior physician actively supported the project and, in doing so, convinced the colleagues.

*“And that serves as a prime example when I have a senior physician who supports the entire [Magnet] project, sees the benefits, and backs it.” (ID13)*

#### 4.5.2. Establishing Practical Relevance

Creating practical relevance was described by interviewees as a crucial factor in making the principles behind the theoretical Magnet model tangible for staff. It was important to show staff specific examples of how Magnet could be applied in their daily work or how they could observe it in action.

*“[…] many employees still have a very abstract idea of it [Magnet] and wonder; what does this mean for me; how can I notice this. But I think with every small step we take and say: ‘By the way, that was Magnet in action’, we’ll improve the reach.” (ID4)*

It became clear, for the interviewees, that linking Magnet to the daily tasks of nursing staff can help increase their acceptance and involvement.

*“Once the connection was made […] to things that already happen in the professional day-to-day or things that need to be addressed, it was relatively easy to get people involved [in the Magnet project].” (ID10)*

The Magnet principle “empirical outcomes” includes the collection and benchmarking of nurse‐sensitive outcome indicators. These were described as an additional way to demonstrate the practical benefits of Magnet. According to one interviewee, this provided nurses with a powerful tool to analyze the quality of care, to assess the impact of patient‐to‐nurse ratios, and to identify strategies for improving patient care.

*“The collection of nurse-sensitive outcome indicators […] could be a game changer in understanding. […] I went through all the clinical areas and introduced the new digital ward management profile, and they were quite shocked. […] But when I explained what it means, what a sharp tool they have in their hands with these numbers, and that they can go to their supervisor and say, look, you took staff away from me, and here are the results. […] See how our numbers have changed.” (ID4)*

#### 4.5.3. Staff Involvement

The active involvement of staff was highlighted as a pivotal factor for the successful dissemination of Magnet. Engaging staff through targeted communication, feedback mechanisms, and participation in training sessions was seen as critical to fostering understanding and acceptance of Magnet.

Interviewees emphasized the importance of using structured formats, such as team meetings and training sessions, as well as informal exchange opportunities to gather feedback and adapt communication strategies. These approaches created multiple touchpoints for staff to voice concerns, seek clarification, and better understand the goals and relevance of Magnet in their daily work environment.

*“We all set off, […] were highly motivated, and at some point, realized that we were deeply immersed in the topics [of Magnet], [that] we understood everything, but the enthusiasm didn’t spread as much as perhaps intended. And this change in perception led to a change in communication. […] we had to take in and accept the feedback from colleagues […]. I think that is crucial for this.” (ID13)*

Regular team meetings served as a platform for fostering dialogue between project teams and staff. Once staff understood the principles and benefits of the Magnet model, they showed more interest and were willing to support the project.

*“In the training sessions and also in everyday exchanges, we […] listened to different opinions. And I always had the feeling that when colleagues truly understood what it [Magnet] was about, they were very, very interested and also had a positive attitude towards it.” (ID10)*

#### 4.5.4. Continuity of Communication

Continuity of communication was repeatedly mentioned as a key factor in the dissemination and acceptance of Magnet. It was seen as essential that the project was communicated regularly and through various media and channels.

*“It’s about continuously staying on track. […] And yes, communicating and doing it again and again. […] It’s like water wearing down stone, slowly but steadily.” (ID4)*

While the interviewee’s metaphor highlights persistence, it was clear across interviews that this continuity was understood not as pressure but as a collaborative process of keeping Magnet visible and meaningful for staff. Regular reminders and open dialogue were seen as ways to maintain engagement and foster shared ownership rather than enforcing change.

It was emphasized that it was important to keep bringing the topic back into focus, as it could quickly be overshadowed in the busy hospital environment.

*“You have to keep, keep, keep reminding people of it, because it often doesn’t stay top of mind in the daily routine.” (ID3)*

Through consistent communication, Magnet became more present and long‐term acceptance was fostered.

*“I think the continuous communication really made the topic [Magnet] more present.” (ID14)*

### 4.6. Navigating Dissemination Challenges

The interviewees identified challenges including difficulties in reaching the target audience and staff resistance.

#### 4.6.1. Difficulties in Reaching the Target Audience

A central challenge the interviewees identified was the difficulty in reaching nurses as the main target group of the implementation of Magnet. This issue was highlighted by several interviewees who emphasized that Magnet had not yet been fully disseminated across the organization.

*“We haven’t managed to spread it [Magnet] widely enough yet, and that’s something we need to focus on in the coming months and years, to really implement it so that it flows into the DNA of the hospital.” (ID1)*

Moreover, there was a lack of experience in identifying which communication channels would be most effective for internal and external communication, and which might need to be addressed first.

*“What we’re still struggling with is the dissemination and communication, both internally and externally, and what this actually means.” (ID3).*

Some interviewees noted that the chosen channels did not reach all employees, requiring a more tailored approach.

*“You always ask yourself; how can we really reach everyone? Not everyone will read the newsletter, not everyone will check social media.” (ID10)*

#### 4.6.2. Staff Resistance

Interviewees reported how resistance and skepticism among staff were significant barriers to successfully disseminating information about Magnet.

One major challenge, one interviewee reflected on, was a lack of understanding or misinterpretation of Magnet, which could lead to less involvement. Some staff were said to be skeptical about the relevance of the initiative to their daily work, while others perceived it as an additional burden rather than an opportunity for improvement.

*“There were also many people who understood and supported it [Magnet], but the lack of understanding led some, I would say, to ignorance and others perhaps even to rejection.” (ID13)*

Additionally, interviewees found it challenging when employees did not openly express their dissatisfaction but reacted more passively.

*“When we talk to individuals, they don’t say to our faces; […]. Instead, they say something like; yes, I think it’s great that we’re doing this, and it’s a great strategy.” (ID7)*

Interviewees also described challenges in communicating and gaining acceptance for Magnet at the leadership level. One interviewee reported skepticism, particularly regarding the leadership role of nurses in the project.

*“And I repeatedly see skepticism from all sides, including leadership, about what [Magnet] is supposed to be and why it is specifically the nurses who are taking the lead. But these are things that need to be discussed and clarified.” (ID1)*

According to the interviewees, a lack of understanding contributed to a passive form of resistance, as some employees did not see themselves as active agents in promoting change. It was reported as challenging to foster a shift in mindset among nurses and to motivate them to actively engage in the implementation of Magnet. Interviewees described a lack of awareness among staff about how to utilize the tools and opportunities of Magnet independently, which was perceived as a barrier to engagement.

*“We are not yet at the point where [Magnet] has fully permeated and everyone knows: ‘I have a problem here, and maybe I can solve it myself using these tools […]’. And for that, we still need a very, very long time to truly achieve enculturation.” (ID3)*

## 5. Discussion

This study explored strategies for communicating and disseminating information in the context of implementing the Magnet principles in German hospitals and contributes to a deeper understanding of communication as a strategic enabler in complex change processes. The findings highlight the use of different communication channels, careful choice of language, and both facilitators and barriers to the dissemination of Magnet, contributing valuable insights for organizational change in healthcare settings.

A broad range of communication channels were identified, encompassing visual, written, and verbal methods implemented both digitally and through in‐person interactions. Tools such as intranet pages and posters provided concise, actionable information to be easily accessible to employees. Ellis et al. [6], who surveyed clinical and nonclinical staff at a hospital undergoing significant transformation, examined the relationships between teamwork culture, communication, change readiness, and burnout. Similar to the findings in the study presented here, they highlighted the importance of using diverse communication types, such as face‐to‐face interactions, emails, and newsletters, to ensure that all staff remains informed and engaged throughout the change process, emphasizing the effectiveness of multiple channels to reach varied audiences [6].

Findings from a literature review by Vermeir et al. [29] on communication in healthcare emphasized the advantages of written communication for conveying information clearly and comprehensibly. At the same time, the review highlighted the importance of direct communication to foster better understanding [29]. In particular, personal interactions such as meetings and training sessions are identified as crucial for sustainably improving communication among healthcare professionals [29]. According to the interviewees in this study, personal interactions played a central role in fostering understanding and engagement with Magnet. Regular team meetings ensured that Magnet remained a visible and prioritized topic, while leadership training integrated these principles into strategic roles, fostering alignment between organizational goals and individual responsibilities. Events, such as celebrations for International Nurses Day, combined recognition of staff with opportunities to raise awareness about Magnet. Findings from a study by Metz et al. [21], who employed a theoretical framework to develop a model for fostering trustful relationships during the implementation of healthcare services, emphasized the importance of structured interpersonal communication in building trust and acceptance during organizational changes. Promoting high‐quality communication strategies that prioritize empathy and positive exchanges can be crucial for enhancing team dynamics and supporting the successful implementation of Magnet.

A key factor emphasized by the interviewees for successful dissemination of Magnet was the role of ambassadors. Acting as multipliers, they bridged communication gaps and promoted engagement among staff. Beyond merely serving as messengers, ambassadors could take on the critical task of adapting Magnet principles to the specific cultural and operational context in a German hospital. By doing so, they ensured that Magnet was not only relatable but also actionable for the staff, to facilitate a smoother integration into existing workflows. This highlights their dual function as cultural translators, as described by Ellis et al. [6], who interpret and contextualize the changes for their colleagues, and as change agents, who actively drive the adoption of Magnet within the organization.

The term “Magnet” was deliberately used in internal communication as a recognizable reference point, helping to structure and align various initiatives under a common vision. This consistency, described as essential for building trust and coherence, allowed staff to associate the term with positive organizational changes and improvements in patient care, fostering acceptance and support. However, this study also emphasized the importance of tailoring terminology to align with local cultural and professional norms. This approach aligns with findings from Svensson et al. [19], who analyzed M4E and found a need to ensure the Magnet principles are accessible and understandable to staff. As the findings of this study suggest, that adaptation of terms is particularly relevant in the German context, especially for those less familiar with English terminology.

Leadership support was identified by interviewees as a beneficial factor in the dissemination process. Leaders who actively advocated for Magnet and motivated their teams created a supportive environment for change. These findings are consistent with those of Kleine et al. [30], emphasizing the critical role of leadership in driving successful change processes. They argue that leaders must not only endorse the vision but also actively communicate with their teams, fostering a sense of urgency and commitment to change [30]. Yet, our results showed that some non‐nursing leaders expressed skepticism towards the nursing‐led nature of Magnet. This highlighted ongoing challenges in communication and a lack of understanding between different hierarchical levels and professional groups, which can hinder the broader acceptance and implementation of Magnet within the organization.

Staff participation and empowerment were seen as facilitators in the dissemination process of Magnet but could also pose barriers if there was resistance from staff. While active involvement of staff, coupled with targeted communication, was essential for fostering engagement, the different ways in which employees received and processed information presented a challenge in developing effective strategies for reaching staff. This reflects insights from the review by Oreg et al. [31], underscoring the importance of transparent and supportive communication that addresses the specific needs and concerns of employees. In this way, resistance can be reduced and acceptance of change increased [31]. Specifically, it was found that trust in the organization and the opportunities for active participation were identified as significantly associated with employees’ support for change [31].

The importance of continuous communication was also noted as beneficial. Regular updates, clear wording, and accessible channels helped address skepticism and resistance. Feedback loops and mechanisms to address staff concerns were critical to maintain engagement, reinforcing findings from Bordia et al. [32] on the value of sustained communication during organizational changes.

Another central aspect was demonstrating the practical relevance of Magnet. Linking abstract concepts to tangible benefits, such as the possibility to improve patient care when measuring nurse‐sensitive outcome indicators, made the principles more relatable and actionable. Practical examples and pilot projects allowed staff to observe real‐world impacts and reduce resistance. This supports the findings of Ellis et al. [6], who identified the importance of connecting change concepts to daily clinical practice to enhance acceptance.

Despite these efforts, challenges in reaching the target audience were also noted. The varied preferences and needs of staff regarding information delivery made it difficult to design communication strategies to reach all target groups. These findings are consistent with those of Mast [7], who highlighted factors such as high workload and the diversity of staff demographics—age, education level, and technological familiarity—complicating communication efforts across various settings. Mast [7] emphasized that managing this complexity requires tailored approaches to effectively reach different employee segments, with younger staff engaging well with digital communication and older staff often preferring face‐to‐face interactions or printed materials.

Finally, resistance and skepticism among employees, particularly nurses, remained significant barriers. These findings reflect known barriers in implementation processes, where perceived complexity, limited understanding, and a lack of perceived relevance can reduce change readiness and engagement [6, 22, 31]. Communication strategies that prioritize transparency, local relevance (i.e., alignment with staff’s specific context, needs, and perceived value), and continuous feedback loops could potentially help address these barriers and strengthen organizational readiness for complex interventions such as Magnet. Similar to the findings of Bordia et al. [32], insufficient understanding and engagement often led to passive or active resistance. Limited communication reach further emphasized the need for innovative and inclusive strategies to involve all staff in the implementation of Magnet.

### 5.1. Strengths and Limitations

A key strength of the study is its focus on the implementation of Magnet in German hospitals, an area that has been underexplored. Most studies on the Magnet model focus on the Anglo‐American context, while limited research addresses implementation challenges in non–English‐speaking countries, including Germany. By applying a qualitative method, the study captures in‐depth, context‐specific perspectives and enables a rich understanding of how communication and dissemination are experienced and interpreted in local implementation processes.

Although the study provides in‐depth insights, its qualitative nature and focus on six hospitals may limit the transferability of the findings to other German hospitals or other healthcare systems due to cultural and organizational differences. However, this context‐specific focus is also what allows the study to reveal detailed insights into the complexity of implementing Magnet principles in Germany, a healthcare setting with limited prior exposure to the Magnet model.

Moreover, the interviews were conducted primarily with individuals in leadership or project coordination positions. This may have excluded important perspectives on communication channels or how information is received by staff at the operational level. Future research should therefore include a broader range of staff perspectives to validate and expand upon the findings. Finally, the use of interviews as the primary data source carries a risk of bias due to social desirability or recall gaps among the interviewees.

## 6. Conclusion

This study sheds light on communication strategies, associated enabling factors, and barriers in implementing and disseminating Magnet in German hospitals. The findings highlight the importance of a multifaceted communication approach that combines visual, written, and personal channels as well as an effective choice of language to reach a wide range of staff. Facilitating factors such as leadership support, continuous communication, establishing practical relevance, and active staff engagement and barriers such as difficulties reaching the target audience, and staff resistance were identified as shaping the communication and dissemination of Magnet.

These insights provide a foundation for hospitals developing strategies to communicate and disseminate the implementation of Magnet and to sustainably embed Magnet in complex hospital environments.

## 7. Practical Implications

Hospitals should use a variety of communication channels, including visual, verbal, and personal interactions, to ensure that Magnet reaches all staff. Effective communication requires careful language choices and the translation of English terms for non‐English countries. Leadership support is central, with leaders acting as role models and emphasizing the initiative’s importance. Engaging staff at all levels, through ambassador teams and feedback, fosters commitment. Hospitals should also emphasize co‐design and ongoing two‐way dialogue so that communication is done with staff rather than to staff. Magnet principles should be clearly translated to facilitate integration into the daily work, with training sessions to clarify benefits. Finally, sustainable communication is essential for driving cultural change and successfully anchoring Magnet in hospital practice.

**Nomenclature**
ANA: American Nurses Association
ANCC: American Nurses Credentialing Center
CNO: Chief Nursing Officer
COREQ: Consolidated criteria for reporting qualitative research
EU: European Union
ID: Interviewee Identifier
M4E: Magnet4Europe
WHO: World Health Organization
U.S.: United States

## Disclosure

The funding sources had no role in study design, data collection and analysis, decision to publish, or preparation of the manuscript.

## Conflicts of Interest

The authors declare no conflicts of interest.

## Author Contributions

Joan Kleine drafted the initial manuscript and was involved in all subsequent steps of data analysis and writing. Joan Kleine conducted the deductive and inductive coding, supported by a student assistant who contributed during the early coding phase. Carolin Gurisch contributed to the analysis discussions. Carolin Gurisch, Julia Köppen, Reinhard Busse, and Lisa Smeds Alenius assisted in finalizing the manuscript. Lisa Smeds Alenius led WP4 of the Magnet4Europe project and played a key role in conceptualizing the conditions under which the interviews were conducted. All authors provided critical feedback and contributed significantly to improving the manuscript.

## Funding

This work was supported by the European Union’s Horizon 2020 Framework Programme (Grant Agreement No. 848031, project: Magnet4Europe–Improving Mental Health and Wellbeing in the Healthcare Workplace). Additional funding for the extended qualitative data collection was provided by the Robert Bosch Stiftung (Grant No. 1000643‐001).

Open access funding was enabled and organized by Projekt DEAL.

## Data Availability

The data that support the findings of this study are available from the corresponding author upon reasonable request.

## Appendix A

The following appendices are provided: Table A1: COREQ‐List and Table A2: Coding tree used for the content analysis. These materials support the methodological transparency and reproducibility of the study.

**Table A1:** Consolidated criteria for reporting qualitative studies (COREQ): 32‐item checklist.

No.	Item	Guide questions/description	Notes	Page number in manuscript
*Domain 1: Research team and reflexivity*				
Personal Characteristics				
1	Interviewer/facilitator	Which author/s conducted the interview or focus group?	15 interviews were conducted by Joan Kleine (9), Julia Köppen (3) and Jutidte Jamal‐Schirmer (3)	—
2	Credentials	What were the researcher’s credentials? E.g., PhD and MD	RN; M.Sc. Joan Kleine (JoK); M.Sc. Carolin Gurisch (CG); RN; M.Sc. Julia Köppen (JuK); Dr. med. MPH FFPH Reinhard Busse (RB); RN; PhD Lisa Smeds Alenius (LSA)	—
3	Occupation	What was their occupation at the time of the study?	JoK, CG, and JuK were research associates and doctoral students. LSA was a researcher and senior assistant lecturer at Karolinska Institutet, SE. RB was a professor of healthcare management at TU Berlin	—
4	Gender	Was the researcher male or female?	Four female, one male	Title page
5	Experience and training	What experience or training did the researcher have?	JoK and CG had academic training and experience of qualitative research as well as 4 years of work experience in this field. In addition, JoK has ten years of work experience as intensive care nurse. JuK has had academic training in qualitative research designs and over 9 years of experience with qualitative and mixed‐methods designs. LSA has extensive experience doing qualitative, mixed methods, and quantitative research. PhD courses in research methods including qualitative research and interviewing techniques. RB is a professor of healthcare management with extensive expertise in comparative health system analysis, health services research, and policy evaluation. He has a medical and public health background and decades of experience in health research, including qualitative and mixed‐methods approaches.	—

Relationship with participants				
6	Relationship established	Was a relationship established prior to study commencement?	JoK and JuK had prior professional interactions with some participants due to their roles in coordinating activities within the Magnet4Europe project, such as learning collaboratives, network meetings, and email communication. These professional interactions with informants facilitated rapport and trust, likely enhancing interview quality, without influencing data collection or analysis	—
7	Participant knowledge of the interviewer	What did the participants know about the researcher? E.g., personal goals and reasons for doing the research	The participants did not know anything about the researchers apart from their professional role in the research project	—
8	Interviewer characteristics	What characteristics were reported about the interviewer/facilitator? E.g., Bias, assumptions, reasons, and interests in the research topic	In each interview the interviewers presented themselves, explained their position at the university and their role in the research	—

*Domain 2: Study design*				
Theoretical framework				
9	Methodological orientation and theory	What methodological orientation was stated to underpin the study? E.g., grounded theory, discourse analysis, ethnography, phenomenology, and content analysis	Interviews were analyzed according to the content analysis by Mayring	7

Participant selection				
10	Sampling	How were participants selected? E.g., purposive, convenience, consecutive, snowball	Participants were selected through purposive and convenience sampling	6
11	Method of approach	How were participants approached? E.g., face‐to‐face, telephone, mail, and email	Participants were sent an invitation letter via e‐mail	—
12	Sample size	How many participants were in the study?	The sample consisted of 16 participants. Two individuals were interviewed together due to time constraints	6
13	Nonparticipation	How many people refused to participate or dropped out? Reasons?	None	8

Setting				
14	Setting of data collection	Where was the data collected? E.g., home, clinic and workplace	Interviews were conducted online and at the participants’ workplaces	6
15	Presence of nonparticipants	Was anyone else present besides the participants and researchers?	No	—
16	Description of sample	What are the important characteristics of the sample? E.g., demographic data and date	The interviewees were Chief Nursing Officers, nursing managers and project managers for the hospital’s Magnet4Europe project engagement	8 (Table 1)

Data collection				
17	Interview guide	Were questions, prompts, guides provided by the authors? Was it pilot tested?	A semistructured interview guide, which was pilot‐tested in advance, was used to conduct the interviews. Interview questions were not provided to the participants in advance	7
18	Repeat interviews	Were repeat interviews carried out? If yes, how many?	Five participants were interviewed for the second respectively third time as part of the Magnet4Europe nested process evaluation. These repeated interviews have likely influenced the nuances and quality of data obtained positively. As for the study presented here, only data from the third round of these repeated interviews are included in the analysis	—
19	Audio/visual recording	Did the research use audio or visual recording to collect the data?	Audio recording with noninternet capable devices was used to collect data	6
20	Field notes	Were field notes made during and/or after the interview or focus group?	Due to audio recording the interviewers did not make notes during the interviews. Lasting impressions were noted after the interviews but not analyzed	—
21	Duration	What was the duration of the interviews or focus group?	Interviews lasted between 37 and 82 min	6
22	Data saturation	Was data saturation discussed?	Sample size was determined using the concept of Information Power [26]	6
23	Transcripts returned	Were transcripts returned to participants for comment and/or correction?	No	—

*Domain 3: analysis and findings*				
Data analysis				
24	Number of data coders	How many data coders coded the data?	Deductive and inductive coding was done by JoK and a student assistant.	7
25	Description of the coding tree	Did authors provide a description of the coding tree?	The complete coding tree is provided in Table A2. Overview of main themes and subthemes is shown in results	7 and (A2)
26	Derivation of themes	Were themes identified in advance or derived from the data?	The identified themes and subthemes were derived from the data.	9–20
27	Software	What software, if applicable, was used to manage the data?	Atlas.ti	7
28	Participant checking	Did participants provide feedback on the findings?	No	—

Reporting				
29	Quotations presented	Were participant quotations presented to illustrate the themes/findings? Was each quotation identified? E.g., participant number	Quotations are used for presenting the results of the study. Each quotation is marked with a participant number	9–20
30	Data and findings consistent	Was there consistency between the data presented and the findings?	Yes	20–24
31	Clarity of major themes	Were major themes clearly presented in the findings?	Four major themes were identified in the analysis, which are named and were used for structuring the results of the interviews. Subthemes were used for subheadings	9–20
32	Clarity of minor themes	Is there a description of diverse cases or discussion of minor themes?	The content of the interviews was presented per theme/subtheme, including the description of diverse/minor cases	9–20

*Note:* Developed from Tong A, Sainsbury P, Craig J. Consolidated criteria for reporting qualitative research (COREQ): a 32‐item checklist for interviews and focus groups. International Journal for Quality in Healthcare. 2007. Volume 19, Number 6: pp. 349–357.

**Table A2:** Coding tree.

Deductive codes (research question)	Inductive codes (subthemes)	Clustered codes (main themes)
Strategies of communicating the implementation of Magnet	Visual and written media	Communicating through multiple channels
	Meetings, team training, and events	
	Use of ambassadors	
	Use of the term “Magnet”	Choosing effective wording
	Tailoring of terminology	

Factors influencing Magnet dissemination	Leadership support	Leveraging enabling factors for dissemination
	Staff involvement	
	Continuity of communication	
	Establishing practical relevance	
	Difficulties in reaching the target audience	Navigating dissemination challenges
	Staff resistance	
